# Hydroxychloroquine Effects on TLR Signalling: Underexposed but Unneglectable in COVID-19

**DOI:** 10.1155/2021/6659410

**Published:** 2021-03-09

**Authors:** Aliede E. in ‘t Veld, Manon A. A. Jansen, Luuk C. A. Ciere, Matthijs Moerland

**Affiliations:** ^1^Centre of Human Drug Research, Leiden, Netherlands; ^2^Leiden University Medical Center, Leiden, Netherlands

## Abstract

The main basis for hydroxychloroquine (HCQ) treatment in COVID-19 is the compound's ability to inhibit viral replication *in vitro*. HCQ also suppresses immunity, mainly by interference in TLR signalling, but reliable clinical data on the extent and nature of HCQ-induced immunosuppression are lacking. Here, we discuss the mechanistic basis for the use of HCQ against SARS-CoV-2 in a prophylactic setting and in a therapeutic setting, at different stages of the disease. We argue that the clinical effect of prophylactic or therapeutic HCQ treatment in COVID-19 depends on the balance between inhibition of viral replication, immunosuppression, and off-target side effects, and that the outcome is probably dependent on disease stage and disease severity. This is supported by the initial outcomes of the well-designed randomized controlled trials: so far, evidence for a beneficial effect of HCQ treatment for COVID-19 is weak and conflicting.

## 1. Introduction

**Figure 1:**
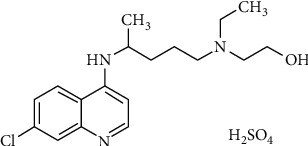
Chemical structure of hydroxychloroquine sulfate.

Hydroxychloroquine sulfate (HCQ, Figure 1) is a less toxic derivative of the antimalarial drug chloroquine (CQ). Besides the use as antimalarial drug, HCQ is also prescribed for the treatment of several different autoimmune diseases such as rheumatoid arthritis, juvenile idiopathic arthritis, and systemic lupus erythematosus. The compound has been evaluated extensively in an ever-increasing number of clinical trials as treatment modality to fight SARS-CoV-2 infection and also in a prophylactic setting. The insight we are aiming to provide in this paper is whether the effects of HCQ use on SARS-CoV-2 infection align with the predicted effects of HCQ. Can the molecular activities of the drug, in particular its direct immunosuppressive activities, predict the effect on SARS-CoV-2 infection? We advocate that especially these immunosuppressive effects ultimately determine the clinical outcome, while so far they have remained largely underexposed in clinical trials evaluating HCQ effects on COVID-19.

The main reason why HCQ initially emerged as potential treatment in COVID-19 was because of its *in vitro* antiviral properties against several RNA viruses, including SARS-CoV-1 and -2 [1–4]. In addition, HCQ possesses immunosuppressive properties that may be beneficial in dampening the derailed immune response in later stages of SARS-CoV-2 infection [5]. Based on these pharmacological activities, HCQ was considered to be a promising drug to combat COVID-19, at least until the moment an effective vaccine would become available. In spring 2020, this even urged some governments to recommend prophylactic HCQ use, for example, by the Indian Council of Medicinal Research [6] and repeatedly by President Trump in White House briefings. This was remarkable, because at that moment in time, conclusive data from large, randomized, and well-monitored clinical trials on the preventive or therapeutic efficacy of HCQ in COVID-19 were pending. The outcomes of initial clinical studies evaluating HCQ effects in COVID-19 were not convincing, also because many studies suffered from major methodological limitations and decent peer review of study reports was complicated by time constraints. This has been extensively outlined in earlier reviews [7] and was breaking news when two papers in The Lancet and New England Journal of Medicine were retracted [8, 9]. Six months later, the results of the first randomized controlled trials have been published, and overall, they are disappointing. So far, there is no convincing proof for beneficial effects of HCQ, neither in a therapeutic setting nor in a postexposure prophylactic setting [10].

A complicating factor for the evaluation of HCQ's effects on COVID-19 has been the highly variable pathophysiology, within an individual patient over time, but also between patients [11]. HCQ's inhibiting effect on SARS-CoV-2 replication, based on *in vitro* evidence, would be beneficial at any stage of the disease, in any population (being it noninfected subjects, asymptomatic patients, or severe patients). However, this is not equally self-evident for the compound's immunosuppressive effects, as we will outline later in this manuscript. Importantly, despite extensive mechanistic evidence based on *in vitro* experiments, reliable clinical data on the extent and nature of HCQ-induced immunosuppression are lacking.

This article discusses the mechanistic basis for the use of HCQ against SARS-CoV-2 in a prophylactic setting and in a therapeutic setting, at different stages of the disease. The focus lies on HCQ's immunosuppressive effects, since we advocate that especially this aspect is largely underexposed in recent clinical trials evaluating HCQ effects on COVID-19. A nonsystematic review of published literature was performed, mainly PubMed-based, to build this mechanistic basis. This article only discusses HCQ, since this compound suffers less from side effects, drug-drug interactions, and toxicity than its parent compound chloroquine, while their pharmacological activities are well comparable [12].

## 2. Immunosuppressive Effects of HCQ

**Figure 2:**
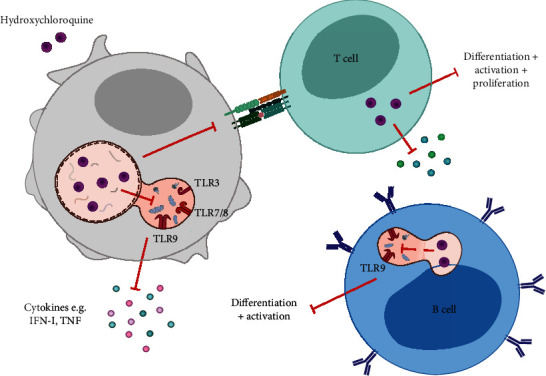
Immunosuppressive effects of HCQ. Hydroxychloroquine affects both the innate and adaptive immune system. By accumulating in the lysosome and autophagosome, the pH is increased causing an inhibition of MHC-II antigen presentation and subsequent T cell activation. In addition, HCQ accumulation abrogates viral recognition by endosomal TLRs, resulting in a decrease of the antiviral innate immune response (i.e., IFN-I production). Moreover, HCQ can also directly affect the adaptive immune system through inhibition of T and B cell differentiation and activation.

The basis for HCQ's use in autoimmune diseases is its wide range of immunosuppressive properties (Figure 2). HCQ accumulates in the lysosomes where it increases the pH and inhibits the enzymatic activity in both lysosomes and autophagosomes. Since these organelles play an important role in antigen processing and MHC class II presentation, a rise in lysosomal pH indirectly inhibits the immune response to both intracellular and extracellular antigens [13].

Lysosomal accumulation of HCQ does not only result in a pH increase, but also directly affect endosomal TLR signalling triggered by nucleic acids. The endosomal TLRs (i.e., TLR3, TLR7, TLR8, and TLR9) play an important role in the innate immune response by recognizing double-stranded RNA, single-stranded RNA, and CpG motifs in viral DNA [14]. HCQ can bind nucleic acids within the endosome, thereby preventing interaction of the endosomal TLRs with their ligands, inhibiting subsequent TLR activation. Downstream innate immune responses are dampened, such as IFN-*α* and TNF production by plasmacytoid dendritic cells [15, 16]. In addition, the adaptive immune response is impaired by HCQ effects on B cell differentiation and cytokine production [17, 18]. Moreover, HCQ inhibits T cell activation, proliferation, and cytokine production by inhibiting intracellular calcium and mobilization and subsequent NFAT signalling [19, 20] and apoptosis in CD45RO+ memory and effector T cells by inhibiting autophagy [21].

The majority of the mechanistic work on HCQ's immunosuppressive activity has been performed in cell lines. Experimental evidence for immune suppression by HCQ in primary human cells is scarce. Some publications are available describing HCQ effects on innate immune responses in human whole blood, peripheral blood mononuclear cells, or T cells, with TLR-mediated cytokine production, or T cell activation and proliferation as endpoint [22–27]. Most experiments used HCQ concentrations largely exceeding expected circulating concentrations *in vivo* after prophylactic or therapeutic dosing. Moreover, with one exception, none of the papers provides a decent HCQ concentration-effect relationship, so an IC50 for HCQ's immunosuppressive activities cannot be estimated. Interestingly, HCQ's IC50 for inhibition of SARS-CoV-2 replication (4-17 *μ*M) [28] appears to exceed HCQ concentrations effectively inhibiting TLR responses *in vitro* (3 *μ*M) [24, 27], which means that it will be difficult to inhibit viral replication without impairing the immune system.

## 3. Mechanistic Support for HCQ Use in COVID-19

### 3.1. Prophylactic Setting

Cell entry by SARS-CoV-2 is thought to be similar to SARS-CoV entry, being mediated by spike (S) protein binding to angiotensin-converting enzyme 2 (ACE2) [29, 30]. *In silico* predictions showed that HCQ prevents the cellular binding and entering of SARS-CoV-2 virus particles, by interfering with sialic acids and surface gangliosides [31]. Based on this pharmacological activity, prophylactic HCQ treatment could theoretically be beneficial and prevent SARS-CoV-2 infection in vulnerable populations or populations professionally exposed to COVID-19 patients.

Upon cell entry, SARS-CoV-2 is likely recognized by TLR3, TLR4, TLR7, TLR8, and RIG-1 [32], resulting in a type I IFN response which is crucial for an efficient adaptive antiviral response [33]. HCQ suppresses parts of the immune system that are essential in fighting infections, including TLR signalling and type I IFN production. In previous SARS-CoV and MERS-CoV outbreaks, downregulation of IFNs by coronavirus proteins strongly correlated with worse disease progression and increased lethality [34]. Cell and animal models of SARS-CoV-2 infection and transcriptional and serum profiling of COVID-19 patients revealed an imbalanced host response with low levels of types I and III IFNs [35]. Early IFN signalling was protective in SARS-CoV-1-infected mice, whereas delayed IFN signalling was detrimental leading to severe disease progression and related lethal pneumonia [36].

The importance of TLR signalling in viral defence has been well established in SARS-CoV-1 mouse models. Both TLR3 and TLR4 deficient mice are more susceptible to SARS-CoV-1 infections [37]. Murine MyD88 or TRIF deficiency, which are downstream signalling molecules shared by multiple TLRs, resulted in a mortality rate of over 90% upon experimental infection with SARS-CoV-1, which is usually nonlethal in immunocompetent mice [37, 38]. HCQ abrogates endosomal acidification thereby reducing endosomal TLR activation [22], but interestingly, enough data confirming this HCQ effect on endosomal TLRs in primary human cells are scarce. Since the relationship between HCQ dose/concentration and level of immunosuppression remains largely unexplored in primary human immune cells, it is difficult to estimate the effect of prophylactic HCQ treatment regimens on the innate immune response. If HCQ's immunosuppressive IC50s would fall in the concentration range reached after prophylactic HCQ treatment, endosomal TLR responses, type I IFN production, and T and B cell activation and proliferation could be impaired *in vivo*. Theoretically, this could result in an increased viral infection risk, including SARS-CoV-2 infection. On the other hand, HCQ use in rheumatoid arthritis patients is not associated with an increased infection risk [39, 40]. So far, prophylactic HCQ studies did not show clinical benefit of HCQ administration [41, 42].

Next to mechanistic arguments, the fact that long-term HCQ use comes with side effects further fuels doubts about prophylactic use of HCQ. Retinal toxicity, cardiac disease, neuromyopathy (reversible), dermatological manifestations, gastrointestinal and hematological changes, and hearing abnormalities have been reported upon long-term HCQ treatment, amongst others [43–45]. Such side effects could be avoided by local HCQ administration, for example, by inhalation.

### 3.2. Therapeutic Setting

Although our understanding of the pathophysiology continues to increase on a daily basis, it is clear that COVID-19 is a highly heterogenous disease. With increased disease severity, the complexity of the pathophysiology grows [32, 46]. Since many excellent reviews are available in the public domain, this manuscript does not revisit COVID-19 pathophysiology and disease progression. Instead, it discusses the alignment between HCQ's mechanism of action and disease stage: how could specific pharmacological activities of HCQ theoretically affect COVID-19's pathophysiology at a particular disease stage? As guidance, the disease progression has been separated into three stages: stage 1—virus entry and replication in the airway cells (days 0-2), stage 2—activation of innate immunity in the lung (maladaptive inflammatory response, days 3-7), and stage 3—acute respiratory distress syndrome (ARDS, >day 7 [47]). Obviously, the clinical presentation of COVID-19 varies between patients from asymptomatic to mild, moderate, and severe, and not all patients develop advanced disease stages.

**Figure 3:**
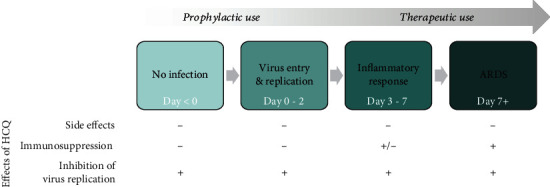
Theoretical effects of HCQ at different stages of SARS-CoV-2 infection. Potential HCQ effects on COVID-19 are schematically presented over the course of the disease, ranging from prophylactic use in uninfected subjects to therapeutic use in acute respiratory distress syndrome (ARDS) in severe patients. A beneficial HCQ effect is indicated with “+” and a detrimental HCQ effect with “-.” The stages of SARS-CoV-2 infection are indicated in green. Stage 0—no infection, stage 1—virus entry and replication in the airway cells, stage 2—activation of innate and adaptive immune system, and stage 3—ARDS.

When discussing potential effects of HCQ treatment in a therapeutic setting, most papers focus on the off-target side effects of HCQ, specifically potentially severe cardiac disorders such as QT segment prolongation. However, safety concerns related to the short-term use of HCQ (i.e., regimens of 1 month) are probably limited, as demonstrated by a recently published (nonpeer reviewed) international study in more than 900,000 HCQ-treated patients [48]. We advocate that one of HCQ's pharmacological activities, namely, its immunosuppressive effect, is critical when considering HCQ as potential treatment modality for COVID-19. Surprisingly, HCQ's exact molecular mechanism of action has remained largely neglected in considerations on therapeutic HCQ use for COVID-19. Therefore, we discuss in the next sections how HCQ's pharmacological activities could be beneficial or detrimental, at different disease stages (stages 1-3, see above) and in different disease severities (asymptomatic, mild, moderate, and severe) (Figure 3).

For therapeutic treatment, the first stage (days 0-2 of infection) is irrelevant, since patients are asymptomatic and viral titers may be low [49], so patients in this stage of the disease are untreated or fall in the prophylactic treatment category (see previous section). HCQ treatment theoretically could be beneficial in the next stages of the disease (stage 2: days 3-7 and stage 3: >day 7), when the innate immune response in the lungs starts to evolve and ultimately culminates in respiratory impairment and multiorgan failure. The drug may not only inhibit virus replication, but also suppress TLR-mediated cytokine responses and overactivation and apoptosis of lymphocytes, processes that are observed in severe COVID-19 [50, 51]. Especially prevention of a cytokine storm is critical since this is a major factor driving multiorgan failure, ARDS, disseminated intravascular coagulation, and the resulting high mortality. Taken together, HCQ treatment in progressed COVID-19 is mechanistically supported by HCQ's pharmacological activities.

Obviously, progressed disease as outlined above (stages 2 and 3) only applies to moderate to severe COVID-19 patients. The large majority of COVID-19 patients only suffers from mild disease or even remains asymptomatic [52]. These patients have a low viral load, develop an efficient type I IFN response, produce virus-neutralizing antibodies, and do not develop a maladaptive inflammatory response [53]. Since it is especially the latter response that could be targeted by HCQ's immunosuppressive activity, the question arises whether HCQ treatment is rational in asymptomatic or mild patients. On one hand, one could argue that HCQ-dependent inhibition of viral replication (though not clinically proven) is important, independent of disease stage. Moreover, HCQ-dependent immunosuppression may prevent mild disease turning into inflammation-driven moderate/severe disease. On the other hand, in the early disease stage, it is important that the virus-specific anti-SARS-CoV-2 response is driven by an efficient antiviral innate immune response, and especially this response may be significantly impaired upon HCQ treatment. The net result of HCQ treatment will depend on the balance between these two pharmacological activities. The outcome of therapeutic studies has shown that HCQ treatment overall does not seem to reduce mortality, improve clinical scores, or suppress viral load in moderate to severe COVID-19 patients [54–56]. However, low-dose HCQ treatment (<2.5 g in total) was associated with a reduced risk of intensive care unit admission and lower mortality rates [57, 58]. HCQ's clinical beneficial effects may depend on the inflammatory status of the patient: chronic low-dose HCQ treatment of a large cohort of rheumatic patients coincided with reduced mortality following SARS-CoV-2 infection [59], and another study reported a therapeutic benefit of HCQ treatment in patients with elevated C-reactive protein levels [60]. These reports are mechanistically in line with the immunosuppressive activities of HCQ, as outlined above.

## 4. Conclusion

Immunosuppression by HCQ, via interference in endosomal TLR signalling, has remained largely underexposed in the public debate, while it may be a critical factor for the (lack of?) clinical efficacy of HCQ in COVID-19. Experimental evidence for immune suppression by HCQ in primary human cells is scarce, which is surprising for such an old drug. Clinical trials evaluating HCQ as COVID-19 treatment did not include readout measures to study this immunosuppressive effect of HCQ. As a result, the extent of immunosuppression by HCQ cannot be reliably estimated *in vivo*. If systemic or local HCQ concentrations would be sufficiently high to suppress key components of the innate immune response, this could translate into a clinical benefit. The other side of the coin is that in mild COVID-19 patients or in a prophylactic setting, immunosuppression by HCQ could have a detrimental effect, since an efficient virus-specific anti-SARS-CoV-2 response depends on a robust antiviral innate immune response. We argue that ultimately the clinical effect of HCQ treatment in COVID-19 depends on the balance between inhibition of viral replication, immunosuppression, and off-target side effects (which have been extensively evaluated recently, within and outside the setting of COVID-19 treatment or prevention, in [61, 62], and are as such not the topic of this article). The outcome of this balance is probably dependent on disease stage and disease severity (Figure 3). This is supported by the initial outcomes of the well-designed randomized controlled trials: so far, evidence for a beneficial effect of HCQ treatment for COVID-19 is weak and conflicting.
