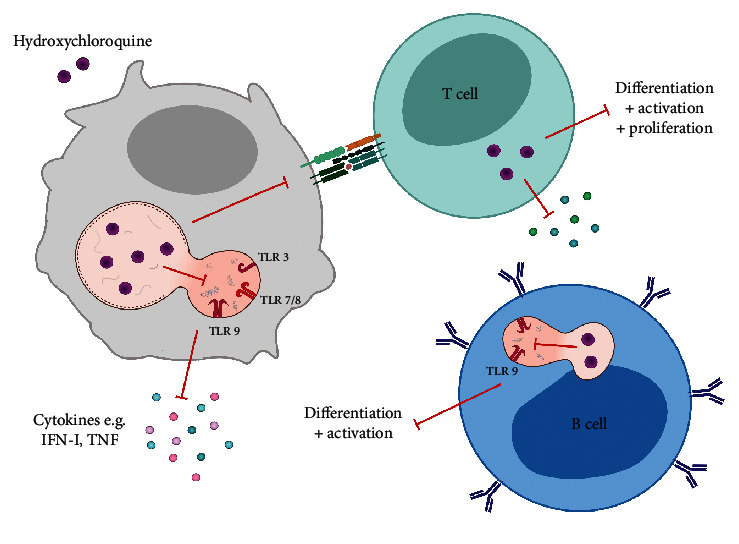# Erratum to “Hydroxychloroquine Effects on TLR Signalling: Underexposed but Unneglectable in COVID-19”

**DOI:** 10.1155/2021/9789246

**Published:** 2021-05-08

**Authors:** Aliede E. in ‘t Veld, Manon A. A. Jansen, Luuk C. A. Ciere, Matthijs Moerland

**Affiliations:** ^1^Centre of Human Drug Research, Leiden, Netherlands; ^2^Leiden University Medical Center, Leiden, Netherlands

In the article titled “Hydroxychloroquine Effects on TLR Signalling: Underexposed but Unneglectable in COVID-19” [1], the incorrect file for Figure 2 was used during the production process and the figure should be corrected as follows:

## Figures and Tables

**Figure 1:**